# Spatially dissecting drivers of inter- and intra-tumoral heterogeneity in Kenyan breast cancer

**DOI:** 10.1186/s13058-026-02347-z

**Published:** 2026-07-29

**Authors:** Mustapha Abubakar, Shahin Sayed, Hela Koka, Scott Lawrence, Karun Mutreja, Xing Hua, Petra Lenz, Samuel Anyaso-Samuel, Rosemary Wangari Kamau, Beryl Akinyi Ooro, Stefan Ambs, Francis Makokha, Li Feng, Jonine Figueroa, Difei Wang, Chad Highfill, Maria Brown, Aaron M. Rozeboom, Kristine Jones, Belynda Hicks, Paul S. Albert, Jianxin Shi, Xiaohong Rose Yang

**Affiliations:** 1 Division of Cancer Epidemiology and Genetics, National Cancer Institute, National Institutes of Health, 9609 Medical Center Drive, Bethesda, Rockville, MD 20850 USA; 2 Aga Khan University, East Africa, Nairobi, P.O Box 30270-00100, Kenya; 3 Cancer Genomics Research Laboratory, Leidos Biomedical Research, Inc, Frederick, MD 21702 USA; 4 AIC Kijabe Hospital, Kijabe, Kenya; 5 Center for Cancer Research, National Cancer Institute, National Institutes of Health, Bethesda, MD 20850 USA; 6 Mount Kenya University, Thika, Kenya

## Abstract

Despite growing evidence on the impact of intra-tumoral heterogeneity (ITH) in breast cancer (BC), its biological drivers and clinically relevant mitigation strategies remain poorly characterized, particularly in low-resource settings where spatial profiling is rarely applied. To address this gap, we conducted a comprehensive, multiplatform spatial profiling study of BC in a Kenyan population. Using the NanoString GeoMx™ Digital Spatial Profiler, we quantified 44 protein markers across 707 spatially defined, PanCK-segmented Areas of Illumination (AOIs) from 31 tumors. All tumors exhibited measurable ITH, with one-third showing marked heterogeneity. Sixteen patients exhibited two or more intrinsic subtypes within their tumors, three of whom had three subtypes. Immune-related ITH was particularly pronounced for CD8 and CD68. AOI phenotype (epithelium- vs. stroma-rich) emerged as the primary driver of marker variability. Linear mixed-effects (LME) modeling further revealed that intrinsic subtype influenced not only classical subtyping markers (ER, PR, HER2) but also immune and signaling markers including IDO1, S100B, PTEN, and BCL-2. Tissue morphology and spatial neighborhood contributed additional variance, particularly in stroma-rich AOIs, while incorporating AOI spatial coordinates improved model fit and revealed further spatially structured heterogeneity. Nonetheless, over 25% residual variability remained for most markers, suggesting unmeasured biological or microenvironmental influences. Comparisons with InSituPlex and bulk RNA data demonstrated that bulk profiling could obscure or misrepresent tumor complexity, underscoring the need for integrative spatial approaches. Our findings highlight whole-slide, multi-region digital profiling as a practical and scalable strategy to capture clinically relevant heterogeneity in low-resource settings.

**Supplementary information:**

The online version contains supplementary material available at https://doi.org/10.1186/s13058-026-02347-z.

## Introduction

Breast cancer is a highly heterogeneous disease at the clinical, population, and molecular levels [1,2]. Based on gene expression profiling or key immunohistochemical (IHC) markers, breast cancer can be classified into at least four major subtypes: luminal A (hormone receptor [HR]-positive, human epidermal growth factor receptor 2 [HER2]-negative, low proliferation), luminal B (HR-positive, and/or HER2 + and/or high proliferation), HER2-enriched, and basal-like or triple-negative (TNBC, estrogen receptor [ER]-negative, progesterone receptor [PR]-negative, and HER2-negative). These subtypes are associated with distinct risk factors, clinical behavior, treatment response, and outcomes. This classification system has significantly improved clinical management and patient outcomes [3]. However, substantial heterogeneity persists within each major subtype in terms of genomic features and clinical outcomes.

Recent research highlights the importance of the breast tumor microenvironment (TME), which consists of diverse cellular components, in shaping tumor evolution and cancer progression through multiple mechanisms such as immune regulation, tissue remodeling, and nutrient supply [4]. This is illustrated by the potential of immune checkpoint inhibitors (ICIs) as an alternative or complementary targeted therapy even when tumor cells lack an actionable target [5]. Consequently, investigating the TME may provide additional clinically and etiologically relevant classifications beyond the traditional breast cancer intrinsic subtypes. For example, characterizing the immune TME in breast cancer patients has revealed significant heterogeneity within luminal and TNBC subtypes [6–8], underscoring the need to account for both tumor and TME heterogeneity when determining optimal therapeutic strategies, including the use of ICIs.

In addition to patient-to-patient differences (intertumoral heterogeneity), tumor and TME cells within an individual patient often exhibit substantial intratumoral heterogeneity (ITH) in morphology, genetic/epigenetic alterations, gene/protein expression, and progression potential [9]. The ITH can be driven by multiple factors, including cell-type(s) of origin, tumor-intrinsic factors, and the interactions between tumor cells and the surrounding TME during multistep tumorigenesis. Single-cell RNA sequencing studies have shown the coexistence of multiple intrinsic breast cancer subtypes within a single tumor [10], which may help explain why some patients are resistant to treatment despite favorable bulk tumor marker profiles. The extent of ITH in subtyping markers, which may impact accurate tumor classification, remains poorly understood. A recent study found that approximately a third of breast cancers demonstrated multiple molecular subtypes within a single tumor and the variations were more noticeable in luminal cancers [11]. Similarly, immune markers within the TME demonstrate spatial heterogeneity, with their predictive value for treatment response and prognosis potentially varying based on spatial context [12–14]. For instance, a study by Carter et al., using the NanoString GeoMx™ platform, revealed distinct immune protein profiles between intraepithelial and stromal areas of TNBC tumors, with patient outcomes linked to the spatial distribution of immune markers [12]. These findings underscore the importance of accounting for ITH when using subtyping and immune markers to guide treatment decisions.

Breast cancer incidence and mortality also vary significantly across populations, with lower incidence but higher mortality rates observed among women of African than European ancestry [15]. These disparities result from biological and non-biological factors, including socioeconomic status, health care access, living environments, and nutrition. Indigenous African populations, in particular, are characterized by diverse genetic architecture and unique environmental exposures, such as higher frequency of protein truncating variants (PTVs) in *BRCA1* and *BRCA2* [16], high burden of infectious diseases, and unique reproductive profiles (high parity and young age at first birth) [17]. These factors may contribute to worse disease outcomes by promoting adverse tumor biology, such as the frequent occurrence of *TP53* mutations and altered immune responses [18,19]. Growing evidence suggests that the distinct biology of the TME may also contribute to more aggressive tumor behavior among women of African ancestry. Compared to women of European ancestry, women of African ancestry tend to have tumors with higher immune infiltration, increased densities of tumor-associated macrophages, endothelial cells, and micro-vessels within the TME [19]. Moreover, the immune cell repertoire has been shown to vary regionally within Africa, with notable differences between West and East African populations [20,21]. Despite these observations, comprehensive investigations on spatial TME in African populations are limited, and the drivers and impact of ITH on tumor and TME classification in women from sub-Saharan Africa remains largely unexplored.

**Fig. 1:**
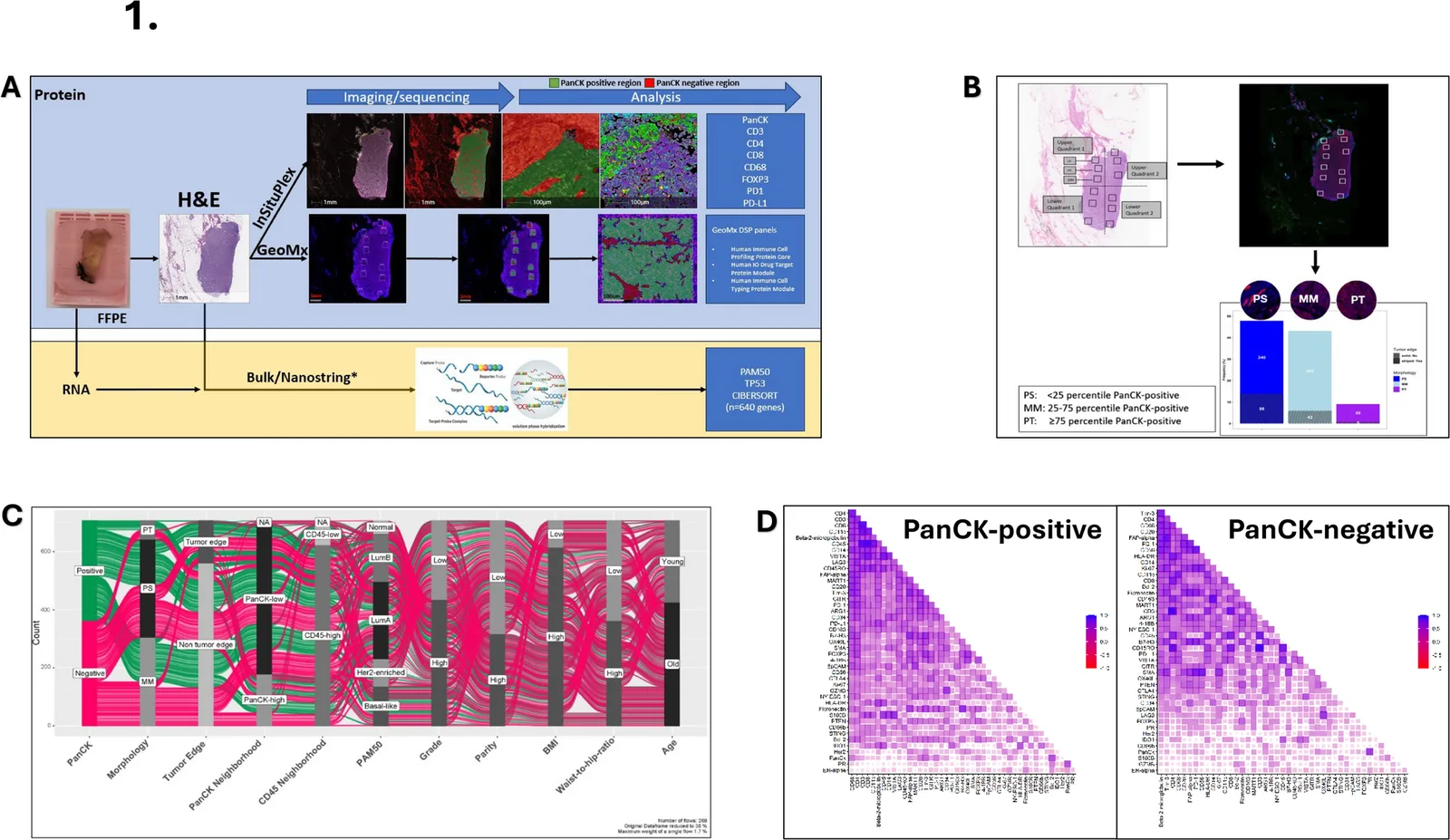
Design of a multiplatform study to investigate intra-tumor heterogeneity in immune and tumor markers of Kenyan breast cancer patients. Panel** A**: Study design of the multiplatform analysis in 31 breast tumors from Kenyan breast cancer patients. Formalin-fixed paraffin-embedded tissue blocks were used to perform hematoxylin and eosin (**H** &** E**) staining and RNA extraction. InSituPlex technology was used for the bulk and spatial analysis of 8 markers using multiplex immunofluorescence staining that included 7 immune markers as well as the Pan cytokeratin epithelial marker. GeoMx digital spatial profiling was used for analyzing 50 markers that included 44 markers of interest as well as 6 control markers. Bulk RNA profiling was performed using the NanoString nCounter technology and immune abundance was estimated using the CIBERSORT algorithm by analyzing 640 genes. Panel** B**: Selection of regions of interest (ROIs) in the GeoMx analysis. Spatial analysis was facilitated by the development of a quadrant approach for whole-slide analysis. Within each quadrant, 3 morphological regions of interest, including regions that are predominantly tumor, mixed morphology, and leading edge were selected. Within each ROI, PanCK immunofluorescence was used to segment epithelial tumor-enriched (PanCK+) and non-epithelial stromal/immune-enriched (PanCK-) AOIs, enabling paired compartment-specific profiling within the same spatial region. In a subsequent step, quantitative data on the amount of PanCK (measured as one of the 44 markers examined) were used to redefine morphological subgroups that include predominantly stromal, PS (< 25th percentile), mixed morphology, MM (25-75th percentile), and predominantly tumor-epithelium, PT (≥ 75th percentile). Panel** C**: The distribution of areas of illumination (AOI) relative to several tissue, tumor, and patient characteristics in this study. Panel** D**: Correlation matrix across 44 markers in epithelium-rich PanCK + and stroma-rich PanCK- regions separately

Therefore, the goal of this study was to comprehensively investigate the drivers and mitigation strategies of ITH in intrinsic subtyping and immune TME markers in breast cancer patients from a Kenyan population. Specifically, we employed the NanoString GeoMx™ platform to quantify 44 key breast cancer intrinsic and immune subtyping markers in spatially defined regions of breast tumors from 31 Kenyan breast cancer patients. These results were compared to data obtained from an in situ staining platform InSituPlex (Ultivue), and bulk NanoString nCounter RNA expression, all generated from the same set of patients (Fig. 1A).

## Methods

### Study population

This study included pathologically confirmed breast cancer patients who were diagnosed at two hospitals in Kenya: Aga Khan University (AKU) Hospital, Nairobi and AIC Kijabe Hospital, Kijabe, between 2019 and 2021. Inclusion criteria were: (1) female sex; (2) pathologically confirmed invasive breast cancer; and (3) availability of adequate formalin-fixed, paraffin-embedded (FFPE) tumor tissue. Exclusion criteria included samples that failed quality control (QC) assessment. FFPE tumor tissue blocks were collected and shipped to the Cancer Genomics Research Laboratory, National Cancer Institute (CGR, NCI). Pathologic characteristics including histologic grade, stage, and ER/PR/HER2 status were extracted from medical records. Immunohistochemical (IHC) staining for ER/PR/HER2 of all breast tumor tissue was done at AKU Hospital, Nairobi. Socio-demographic, clinical, reproductive, and known breast cancer risk factor data were collected using a standardized questionnaire as previously described [22].

ER and PR tumor expression were considered positive by IHC with ≥ 1% nuclear staining based on the ASCO/CAP guidelines [23]. HER2 expression was predominantly determined by IHC and Fluorescence in situ hybridization (FISH) data was available for a subset of patients. An IHC score of 3 + or a FISH positive test result was defined as HER2-positive. We used Ki-67 status (low defined as < 20% and high defined as ≥ 20%) to discriminate luminal A and B and used tumor grade as a surrogate for patients with missing Ki-67. Molecular subtypes based on IHC surrogates were defined as: luminal A: ER + and/or PR+, HER2−, and low Ki-67/histologic grade (I or II); luminal B-HER2+: ER + and/or PR+, and HER2+; luminal B-high proliferative: ER + or PR+, HER2−, and high Ki-67/histologic grade (III); HER2-enriched: ER−, PR−, and HER2+; and triple-negative (TN): ER−,PR−, HER2−.

This study was conducted within a larger ongoing hospital-based breast cancer study in Kenya, from which FFPE tumors have been collected from approximately 120 patients to date. Patients were included in the present analysis if they had sufficient tissue and complete analyzable data across all three platforms used in this study: GeoMx DSP, Ultivue/InSituPlex imaging, and NanoString nCounter bulk expression profiling. Additional requirements included adequate FFPE tissue for selection of multiple ROIs representing the intended histologic and spatial contexts (see the section below), as well as successful completion of platform-specific QC steps. A total of 31 patients were selected, who had an ER-positivity distribution broadly similar to that of the larger source cohort (**Supplementary Table 1**).

### GeoMx DSP assay

Two serial whole-tissue sections were created from the FFPE block of each patient: one was stained with H&E and the other was stained for GeoMx-DSP protein markers using the standard protocol recommended by the manufacturer (NanoString Technologies Inc., Seattle, WA) [24]. H&E slides were reviewed by the study pathologists (MA and PL) using the HALO system (Indica Labs, Albuquerque, NM). To capture spatial heterogeneity across each tumor while remaining within the throughput constraints of the GeoMx DSP platform, 12 regions of interest (ROIs) were selected based on the pathologist’s qualitative assessments of tissue morphological characteristics [e.g., predominantly tumor, predominantly stroma, or mixed morphology], as well as spatial location (such as ROIs located near the invasive tumor leading edge), within the tissue. In general, 3 representative ROIs with different morphology were selected in each of the four tissue quadrants (Fig. 1B, top left panel). Because the GeoMx DSP workflow requires ROIs to be defined before molecular segmentation, these ROIs were intentionally selected to sample distinct tissue microenvironments expected to differ biologically, including tumor-rich regions, stroma-rich regions, mixed epithelial-stromal regions, and areas adjacent to the invasive tumor front. ROI selection was intended to provide representative spatial sampling across the tissue rather than to define the final biological units for analysis. To further ensure representative sampling across the entire tissue section, the specimen was divided into four quadrants and, in general, three representative ROIs with different morphologies were selected from each quadrant (Fig. 1B, top left panel). This approach reduced spatial clustering of ROIs and increased coverage of geographically distinct tumor microenvironments across the tissue section. Two pathologists were involved in ROI selection and review, with one pathologist (MA) initially annotating candidate ROIs representing prespecified histologic/spatial categories and the second pathologist (PL) reviewing these candidate ROIs, refining the histologic criteria, and selecting the final ROIs based on histologic representativeness and technical suitability for GeoMx DSP. Annotated H&E images were overlaid on GeoMx scans to transfer the ROIs created by the pathologists. The following protein marker modules were used for the GeoMx assay (Fig. 1B, top right panel): Human Immune Cell Profiling Protein Core, Human IO Drug Target Protein, Human Immune Cell Typing Protein, Human Pan-Tumor Protein, and Human solid Tumor TME Morphology, which included 50 different proteins (44 targeted markers + 6 control markers, **Supplementary Table 2**). The slides were visualized using a NanoString GeoMx DSP instrument. The GeoMx morphology marker panel includes immunofluorescent staining for pan-cytokeratin (PanCK), which was used to identify epithelial tumor cells and digitally segment each ROI into epithelial (PanCK+) and non-epithelial (PanCK−) AOIs (Areas of Illumination). Thus, each ROI generated paired PanCK + and PanCK− AOIs when both compartments were present. Protein expression was quantified separately within each AOI, and these AOIs constituted the primary analytical units used for all downstream analyses. This design allowed us to evaluate both regional heterogeneity across tumor microenvironments and compartment-specific marker expression within the same histologic region. We conducted the following QC steps. First, we calculated the hybridization factors and removed any sample with a value ≥ 10. ERCC normalization was then performed using the hybridization factors. Next, we plotted nuclei count and AOI area and removed AOIs with < 25 nuclei and < 1000 µm^2^ AOI area. Among the three housekeeper proteins, Histone H3 and S6 displayed the highest correlation and least amount of noise, so we used these two proteins to perform normalization on the data and removed AOIs with large normalization factors (log NF > 5). In total, 719 AOI segments were profiled, of which 707 passed QC criteria and were included in the downstream analysis.

We calculated the signal to noise ratio (SNR) of each protein marker using the geometric mean of the IgGs. SNR was above background for most markers (**Supplementary Fig. 1**), and we did not filter proteins based on SNR. However, we treated markers with low SNRs across AOIs with caution in the downstream analyses. From the initial set of proteins analyzed, background immunoglobulin G (IgG) controls and housekeeping proteins were removed, and 44 targeted proteins were retained for further evaluation.

The annotation of AOI morphology based on visual ROI selection was subjective and prone to differences across patients. For example, predominantly tumor regions in one tumor may be classified as mixed morphology in another tumor due to different cell composition in different tumors. To standardize the morphologic classification across all AOIs and patients, we used quantitative PanCK expression measured by GeoMx to redefine the morphology of each AOI as follows: predominantly tumor-epithelium (defined as ≥ 75 percentile PanCK+ area across all AOIs), mix-morphology (25–75 percentile PanCK+ area), and predominantly stroma (< 25 percentile PanCK+ area) (Fig. 1B, bottom panel). These quantitative PanCK-based morphology labels were used in all downstream analyses.

To assess ITH of subtyping markers, we assigned AOI-specific subtypes based on the marker expression levels of ER-alpha, PR, and HER2 among PanCK+ (epithelium-rich) AOIs. Each AOI was classified as negative or positive for each marker after identifying an optimal cut-off point that maximized the separation between groups defined by the IHC status of the corresponding marker. In this process, we aimed to optimize the sum of sensitivity and specificity to find the most effective distinguishing point. The subtypes were defined as follows: Luminal A: ER + and/or PR+, HER2−; Luminal B: ER + and/or PR+, HER2+; HER2-enriched: ER−, PR−, HER2+; and Triple Negative: ER−, PR−, HER2−.

### Multiplex immunofluorescence staining and imaging analysis

Using the InSituPlex approach, an 8-plex immune-profiling panel was designed and tested to detect and classify T cells, macrophages, and tumor cell populations along with PD-L1 checkpoint expression in multiple tumor types. The assay was run on multiple FFPE tissue samples from the same set of 31 breast tumors that were analyzed using GeoMx. Each sample was stained for the following eight markers: CD3, CD4, CD8, CD68, FOXP3, PD-1, PD-L1, and PanCK. Staining was performed in a single step using the Leica Biosystems BOND RX autostainer. The whole-slide imaging (WSI) was performed in two rounds on the ZEISS Axio Scan.Z1 slide scanner. 4-plex image (excluding DAPI) generated in each round was then stacked using the Ultistacker software (Ultivue, Cambridge, MA) to create the 8-plex image.

Identification of individual cells and phenotypes was performed using cell segmentation and classification tools implemented in HALO (Indica Labs). In the first step a custom nuclei segmentor was created using the default Nuclei Segmentor provided by HALO, module name: Nuclei Seg (HALO AI). Following that, three classifiers were created using the DenseNet module of HALO: (1) a classifier to distinguish between tissue, glass and adipose tissue; (2) a classifier to distinguish between positively and negatively stained PanCK regions; and (3) a classifier to mark red blood cells in the tissue. An algorithm was then developed using HALO’s highplex FL module (Indica Labs- HighPlex FL v4.0.4) which incorporated the custom nuclei segmentor and the RBC classifier. The algorithm was run on the tissue region generated by the first classifier and PanCK positive and PanCK negative regions generated by the second classifier. Pathologists were consulted to annotate the negative regions for each marker. Marker thresholds in the algorithm were validated by comparing cell intensity in positive control and the negative regions. A cell was considered positive if it had expression levels above the threshold for both DAPI (as the nuclear marker) and the corresponding cell specific marker.

AOI neighborhoods were identified by exporting the GeoMx defined AOIs to the GeoMx slide images (stained for PanCK and CD45 as per the GeoMx assay) into the HALO image analysis platform. For each GeoMx AOI the regions were expanded 500 μm from the primary AOI. Using the Area and CytoNuclear modules for PanCK and CD45 respectively, the amount of PanCK and CD45 was quantified for each AOI neighborhood.

### Nanostring bulk expression analysis and intrinsic subtype calling

All 31 tumor blocks were processed at CGR, NCI. Total RNA was extracted using Qiagen kit from FFPE tissue blocks that were processed to enrich tumor cells through macro-dissecting the tumor region and quantitated using the Qubit RNA Broad Range Assay (ThermoFisher, Waltham, MA). One hundred ng of total RNA for each sample was normalized to 5ul. Hybridization of the sample to the nCounter custom codeset (NanoString, Seattle, WA) that included gene sets for PAM50, and immune-related genes implemented in CIBERSORTx was performed according to the manufacturer’s protocol (MAN-10056-05) for nCounter XT CodeSet Gene Expression Assays. After hybridization, samples were further prepared and immobilized onto cartridges using the NanoString nCounter Prep Station 5s. The cartridges were then scanned on the nCounter Digital Analyzer, according to the manufacturer’s protocol. NanoString nSolver software was used to perform preliminary QC of the sample data. All 31 samples passed the quality check and were normalized using nSolver. PAM50 subtype (luminal A, luminal B, HER2-enriched, basal-like, and normal-like) was defined by an absolute intrinsic subtyping (AIMS) method using NanoString data at patient level [25].

### Statistical analyses

For each marker, we performed a linear mixed-effects (LME) model (referred as the M1 model)

$$ {y} = {X}_{1} {\beta } + {X}_{2} {\gamma } + {\mu } + {\eta }_{{spatial}} + {\varepsilon }, $$.

where $X_{1}$ represents fixed effect covariates at subject level (age, PAM50 subtype, whole tissue area), $X_{2}$ represents fixed effect covariates at AOI level (ROI morphology, ROI neighborhood, AOI segmentation (PanCK+/-). The term $\mu \sim N\left( {0,\sigma _{1}^{2} } \right)$ denotes the subject-specific random effect, ${\eta }_{{spatial}} \sim N\left( {0,\sigma _{\eta }^{2} {R}} \right)$ captures spatially correlated residuals with $R_{{ij}} = \exp \left( { - d_{{ij}}^{2} /\phi ^{2} } \right)$, where $d_{{ij}}$ is the Euclidean distance between spatial coordinates of the two AOIs from the same subject, and ${\varepsilon }\sim N\left( {0,\sigma _{0}^{2} I} \right)$ is the independent unstructured residual errors. LME models were fitted using function *lme4* in the *nmle* package in R. Confidence intervals were calculated using the *intervals* function from the same package.

The total phenotypic variance can be decomposed as follows:

$$ var\left( y \right) = \sigma _{b}^{2} + \sigma _{w}^{2} = \left( {\tau _{1}^{2} + \sigma _{1}^{2} } \right) + \left( {\tau _{2}^{2} + \sigma _{\eta }^{2} + \sigma _{0}^{2} } \right), $$

where the inter-subject variance $\sigma _{b}^{2}$ is decomposed to the variance $(\tau _{1}^{2} )$ that can be explained by covariates at the subject level and the unexplained variance $\sigma _{1}^{2}$; the between-subject variance $\sigma _{w}^{2}$ is decomposed into the variance $\tau _{2}^{2}$ that can be explained by covariates of AOI characteristics, the spatial residual variance $\sigma _{\eta }^{2}$, and the independent residue variance $\sigma _{0}^{2}$. Notably, the between-subject variance $\sigma _{w}^{2}$ represents the intra-tumoral heterogeneity (ITH). Based on this modelling framework, ITH is further decomposed to fixed-effect ITH (explained by AOI characteristics), spatially structured ITH, and unexplained ITH. Moreover, we also fit a reduced linear mixed-effect model without explicitly modelling the spatial-related random effect ${\eta }_{{spatial}}$, referred to as M0 model.

We performed clustering analysis of all 707 AOIs based on Euclidean distance calculated from z-statistics. Clustering analysis was performed using the function Heatmap.3 as implemented by the package *gplots* in R (https://cran.r-project.org/package=gplots*).* All visualization materials were generated using R version 3.2.0 (R Foundation for Statistical Computing, Vienna, Austria).

## Results

### Characteristics of the study population

We conducted GeoMx DSP (Digital Spatial Profiling), InSituPlex, and NanoString nCounter bulk expression analyses on tumors from 31 treatment-naive Kenyan breast cancer patients (Fig. 1A). Baseline demographic and clinicopathological characteristics of these patients are described in **Supplementary Table 1**. Briefly, the mean age at diagnosis was 51.2 years old (range 20.0–72.0 years). The distribution of intrinsic subtype, determined by PAM50 from bulk expression (see Methods), was luminal A (38.7%), luminal B (22.6%), HER2-enriched (12.9%), basal-like (19.4%), and normal-like (6.5%). The majority of these patients (61.3%) had high grade tumors.

### Digital spatial profiling of Kenyan samples reveals inter- and intra-tumor heterogeneity

For the GeoMx DSP analysis, as described in the Methods section, we initially selected 12 regions of interest (ROIs) from each tumor based on morphological characteristics, including tumor, stroma, or mixed tumor-stromal regions, and their spatial distribution (Fig. 1B). To standardize the morphologic classification across all ROIs and patients, we used PanCK expression measured by GeoMx (PanCK was one of the 44 markers examined) to categorize each ROI into three morphological groups: predominantly tumor-epithelium (defined as an ROI comprising ≥ 75 percentile PanCK+ area), mix-morphology (ROI consisting of 25–75 percentile PanCK+ area), and predominantly stroma (ROI comprising < 25 percentile PanCK+ area) (Fig. 1B).

Immunofluorescence (IF) staining of PanCK was employed as a phenotypic marker to further classify each ROI into two distinct areas of illumination (AOIs): PanCK+ (epithelial) and PanCK- negative (non-epithelial i.e., stromal) areas. This approach yielded 24 AOIs per patient. After excluding AOIs that failed quality control (QC) steps (see **Methods**), 707 AOIs passed QC and were included in downstream analyses.

We analyzed the distribution of breast cancer risk factors and clinicopathological characteristics, including patient-level factors (age, parity, BMI, hip-to-waist ratio, PAM50 subtype, and tumor grade) and AOI-level features (epithelial vs. stromal, morphologic group [predominantly tumor-epithelium, predominantly stroma, mixed morphology], tumor leading edge [yes or no], and AOI neighborhood [see **Methods**, PanCK/tumor-enriched or CD45/leukocyte-enriched neighborhood]) (Fig. 1C). By design, the proportions of epithelial (PanCK+) and stromal (PanCK-) AOIs were balanced across the samples. Overall, most of the AOIs were predominantly stroma or mix-morphology, with fewer being predominantly tumor. As would be expected, PanCK+ AOIs predominated in PanCK+ neighborhoods while PanCK-AOIs predominated in CD45-enriched neighborhoods (Fig. 1C).

**Fig. 2:**
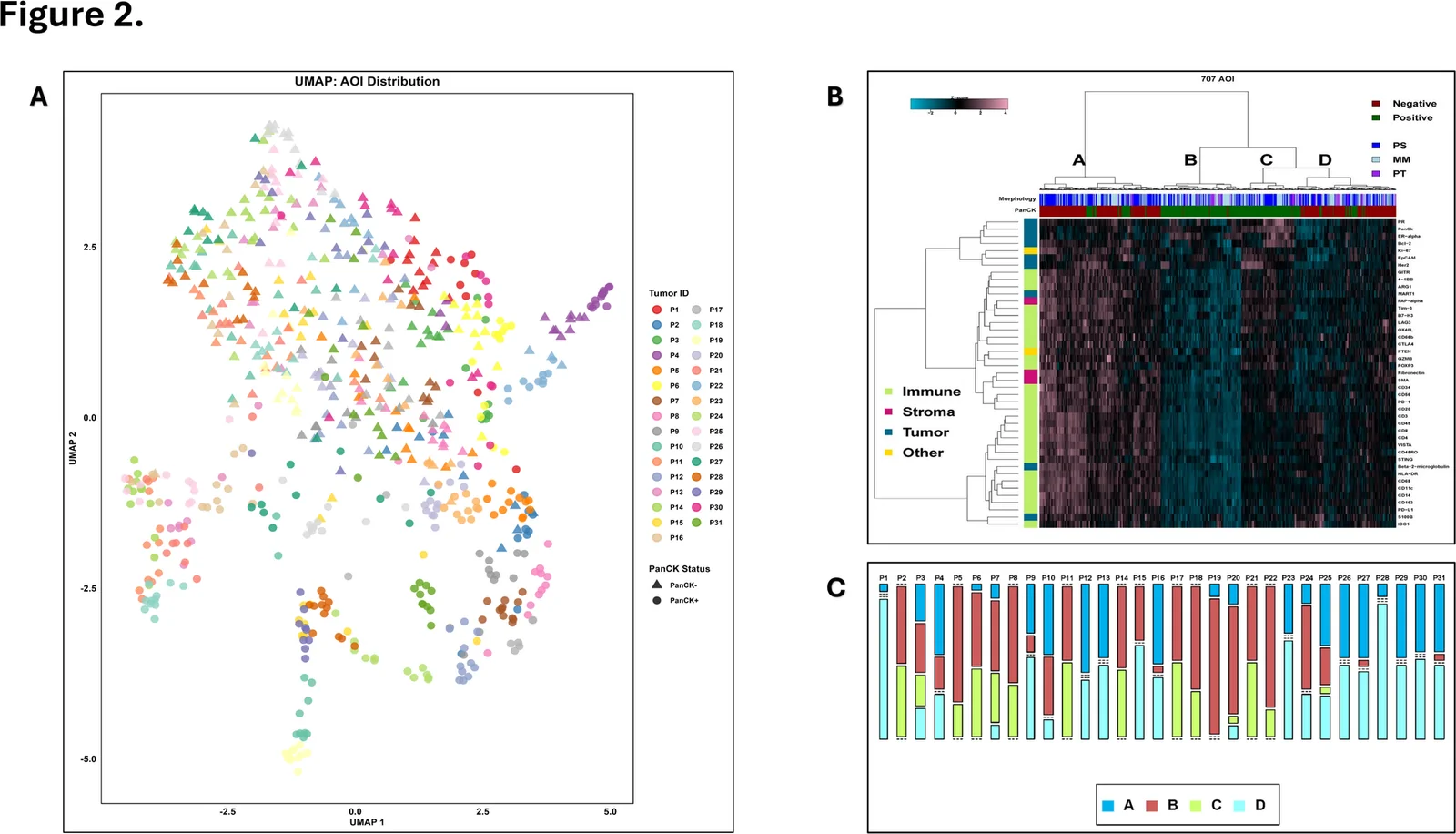
Clustering of 44 protein markers across 707 Areas of Illumination (AOIs). Panel** A**: Clustering of AOIs based on expression of 44 markers using Uniform Manifold Approximation and Projection (UMAP) (n_neighbors = 30, min_dist = 0.3, metric = Euclidean, and n_components = 2). Panel** B**: Consensus clustering of 44 protein markers across 707 AOIs identified four clusters. Panel** C**: Distribution of clusters across 24 AOIs within each tumor sample

The correlation matrix of 44 measured protein markers, most of which are immune-related TME markers (**Supplementary Table 2**), is shown in Fig. 1D, for PanCK + and PanCK- AOIs separately. Using Uniform Manifold Approximation and Projection (UMAP) to visualize marker expression patterns across all AOIs, we observed a separation primarily driven by AOI phenotype (PanCK status). AOIs originating from the same tumor also tended to cluster together, indicating that intertumoral heterogeneity exceeded intratumoral variability (Fig. 2A). Based on the expression profiles of these 44 protein markers, consensus clustering analysis divided the 707 AOIs into four clusters (Fig. 2B). These clusters were primarily segregated by AOI phenotype (PanCK + vs. PanCK-) and ER-alpha status. Clusters B and C were enriched for epithelium-rich AOIs, while clusters A and D were enriched for stroma-rich AOIs. Cluster D was enriched with AOIs that expressed lower levels of ER-alpha compared with AOIs in other clusters. Immune and stromal markers exhibited the highest expression levels in cluster A tumors and the lowest in cluster B tumors, suggesting that TME markers could enhance subtype classification beyond epithelial marker-based stratifications. Figure 2C illustrates the cluster assignments for each patient across all AOIs. All 31 tumors exhibited some degree of ITH, with AOIs within a single tumor often assigned to multiple clusters. Notably, 12 tumors had AOIs distributed across three or more clusters, highlighting substantial ITH. For instance, patient P3 had a similar number of AOIs assigned to each of the four clusters (Fig. 2C).

### Drivers of inter- and intra-tumor heterogeneity of tumor and TME markers in tissues

**Fig. 3:**
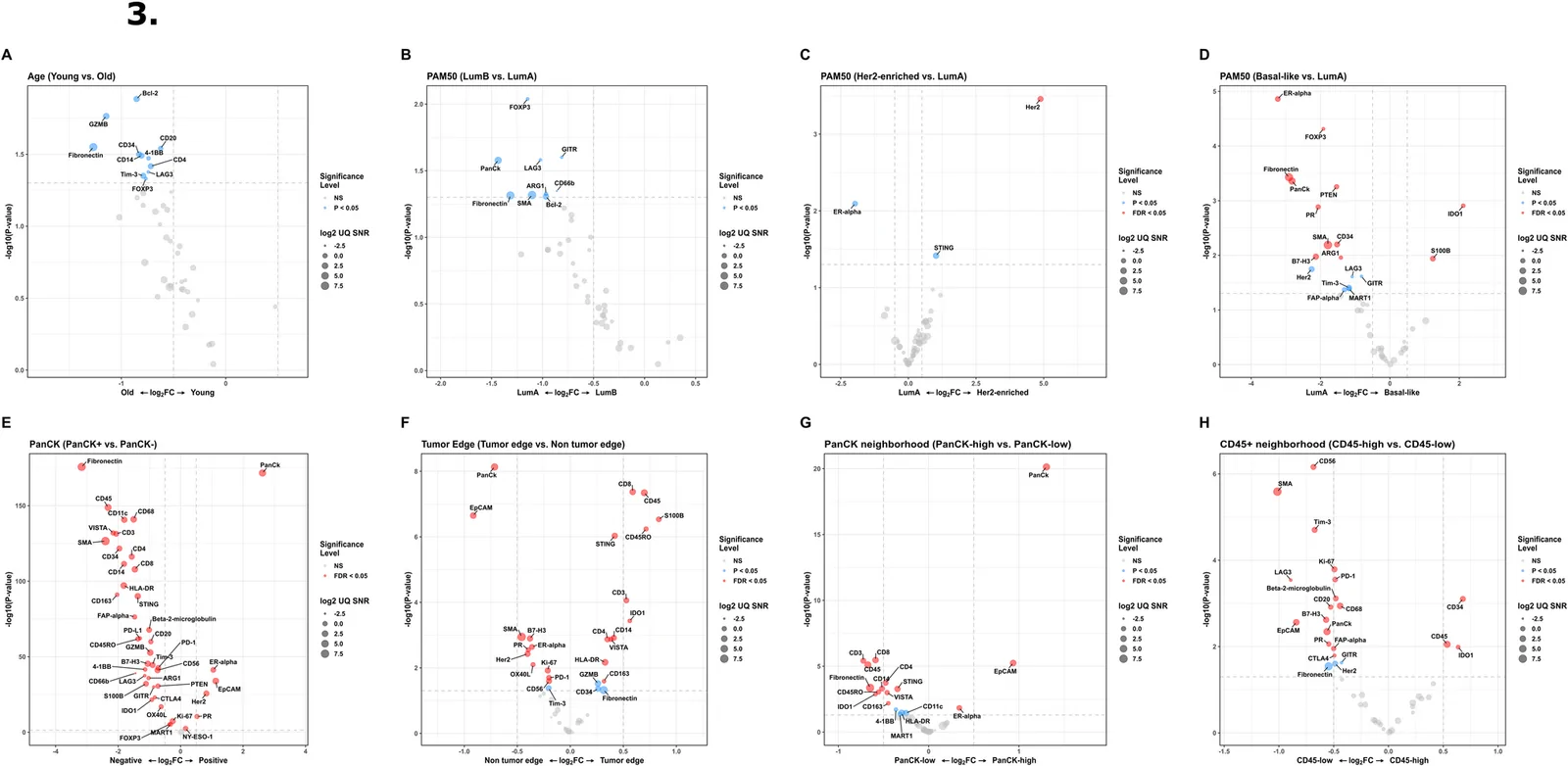
Differential marker expression by patient and AOI-level characteristics. Panel** A**: patient age (young vs. old); Panel** B**: PAM50 subtype (Luminal B vs. Luminal A); Panel** C**: PAM50 subtype (HER2-enriched vs. Luminal A); Panel** D**: PAM50 subtype (Basal-like vs. Luminal A); Panel** E**: AOI phenotype (PanCK + vs. PanCK-); Panel** F**: tumor leading edge (vs. non-tumor edge); Panel** G**: PanCK neighborhood (high vs. low); and Panel** H**: CD45 neighborhood (high vs. low)

Having demonstrated the presence of inter- and intra-tumoral heterogeneity in these samples, we sought to address our key aim of characterizing their drivers. To achieve this, we evaluated the impact of patient-level and AOI-level characteristics on the protein expression of 44 markers using differential expression analysis and linear mixed-effects (LME) models and the results are as follows:

*Patient-level characteristics*: A few markers showed nominal variation by some patient-level characteristics such as age (e.g., Bcl-2 and GZMB, Fig. 3A) and grade (e.g., Fibronectin, PTEN, and FOXP3, **Supplementary Fig. 2**), but differences generally did not reach statistical significance after correcting for multiple comparisons, which might be due to the small sample size for patient-level comparisons (**Supplementary Table 3**). As expected, ER-alpha, PR, and HER2 expression varied significantly across intrinsic subtypes (Fig. 3B–D). Additionally, AOIs in basal-like tumors showed higher IDO1 and S100B expression levels and lower levels of PanCK, CD34, FOXP3, and PTEN compared to AOIs in luminal A tumors (FDR < 0.05, **Supplementary Table 3**, Fig. 3D).

*AOI-level characteristics*: We found tissue morphology to be associated with ITH in expression of most markers. In analyses comparing PanCK+ (epithelium-rich) versus PanCK- (stroma-rich) AOIs, we observed differential expression of all markers (Fig. 3E). Twenty-eight markers were also significantly differentially expressed according to tissue morphology (i.e., predominantly tumor-epithelium, mix-morphology, predominantly stroma; FDR < 0.05), with the majority being stromal or immune markers (**Supplementary Table 3**,** Supplementary Fig. 2)**. Most of these markers displayed higher expression levels in mix-morphology or predominantly stroma compared to predominantly tumor-epithelium AOIs, except for GITR, CD34, CD66b, and EpCAM, which had lower expression in mix-morphology/predominantly stroma compared to predominantly tumor-epithelium AOIs. Six markers were differentially expressed in the tumor leading edge as compared to the non-leading-edge areas, including S100B, CD45R0, CD45, STING, and CD8 (Fig. 3F). Additionally, the expression of some markers, e.g., EpCAM, ER-alpha, TIM3, Ki-67 also varied by AOI neighborhood, i.e., whether the AOI was located in a PanCK+/- or CD45-low/high neighborhood (Fig. 3G and H). Some markers (e.g., ER-alpha and CD8) showed a synergistic relationship between an AOI and its corresponding neighborhood, indicating coordinated regulation between local and adjacent cellular contexts, while some others demonstrated a potentiating relationship. For example, SMA expression was higher in stroma-rich AOIs located within PanCK+ neighborhoods than in those within PanCK- neighborhoods (**Supplementary Fig. 3**), suggesting a potential tumor-stroma interaction that influences myofibroblast deposition.

*Heterogeneity due to measured and unmeasured factors*: We further examined the variance in marker expression explained by patient and AOI characteristics obtained from LME models with the mutual adjustment of all examined variables and accounted for the lack of independence among AOIs within a single patient. Because PanCK status strongly influenced most markers, analyses were conducted separately for PanCK+ (epithelium-rich) and PanCK- (stroma-rich) AOIs.

**Fig. 4:**
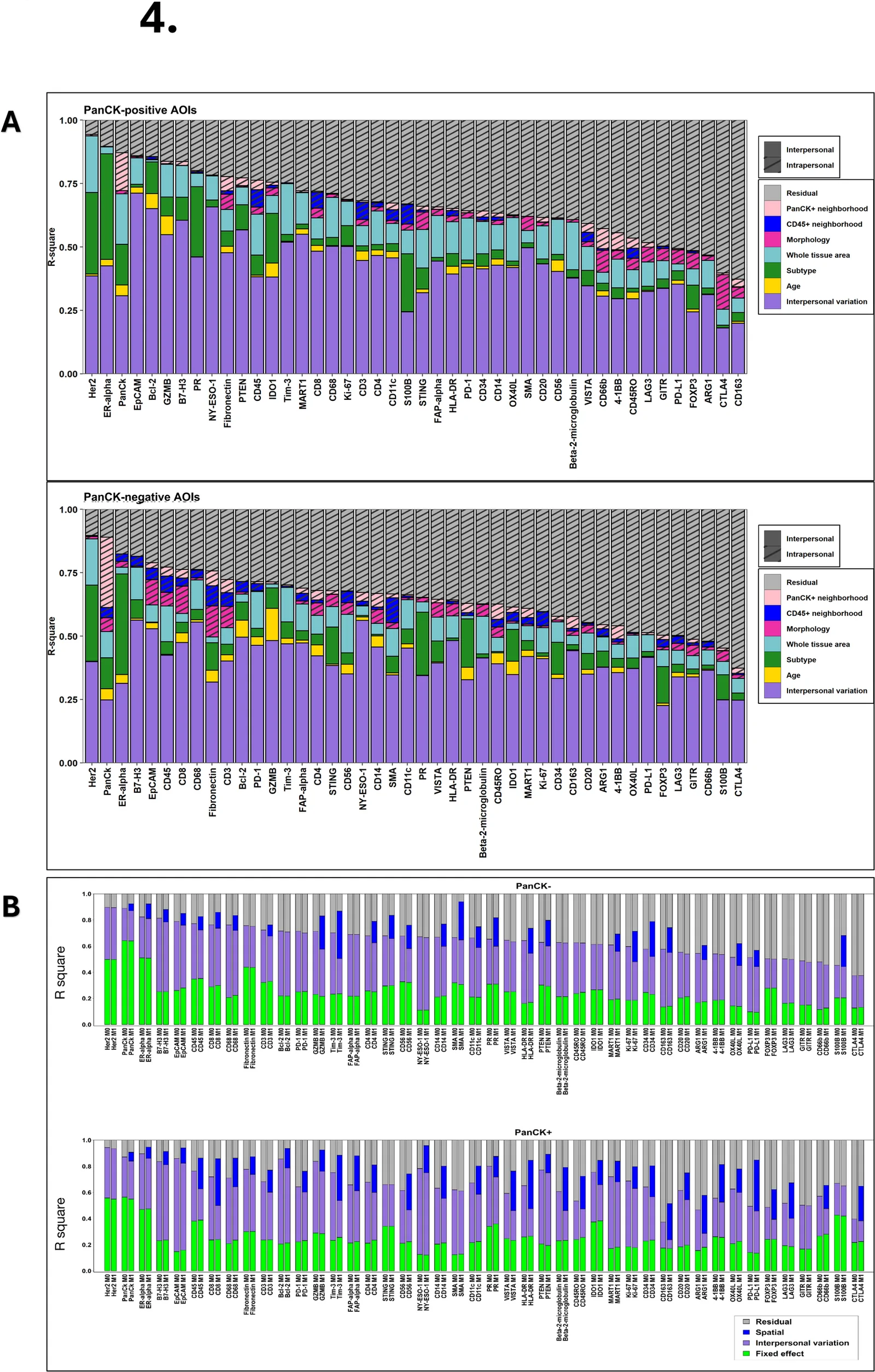
Contribution of patient and AOI-level characteristics to variance of marker expression. Panel** A**: The proportion of total variance in each marker explained by (1) intertumoral random effects, (2) fixed effects of known covariates including both patient and AOI-level characteristics, and (3) residual intratumoral effects in M0 LME models among PanCK+ (top panel) and PanCK- (bottom panel) AOIs. Panel** B**: The contribution of a spatial variable (AOI coordinates) to the variance of marker expression. M0 model: LME model without AOI coordinates. M1 model: LME model with AOI coordinates. Fixed effects of known covariates are summarized and presented together (indicated by green bars)

Figure 4A shows the proportion of total variance in each marker explained by (1) intertumoral random effects, (2) fixed effects of known covariates hypothesized to influence intertumoral and intratumoral heterogeneity, and (3) residual intratumoral effects in the M0 model (see **Methods**). Across both PanCK + and PanCK- AOIs, a large share of the variance was explained by interpersonal random effects (Fig. 4A), underscoring patient-to-patient variability. Among fixed effects, PAM50 subtype and whole tissue area were the strongest contributors for most markers. Age effects were modest, except for GZMB (particularly in PanCK-AOIs) and, to a lesser extent, BCL-2.

Within PanCK+ AOIs, not surprisingly, the majority of variance in ER, PR, and HER2 expression was explained by the PAM50 subtype. Additionally, PAM50 also explained a sizable proportion of variance in the expression levels of IDO1, S100B, and BCL-2 (Fig. 4A). Even among PanCK- AOIs, PAM50 subtype remained a key contributor for a few markers such as PTEN, IDO1, CD34, FOXP3, STING, fibronectin, and GZMB, in addition to subtyping markers (ER, PR, and HER2) (Fig. 4A). AOI-level variables such as tissue morphology, PanCK/CD45 neighborhood explained more variance in PanCK- than in PanCK+ AOIs. In stroma-rich PanCK- AOIs, tissue morphology contributed most strongly to EpCAM, CD3, CD8, and fibronectin, while CD45 + neighborhood effects were prominent for fibronectin, SMA, and Ki-67.

As shown in Fig. 4A and **Supplementary Table 3**, whole tissue area (the area of whole tissue section for each tumor where AOIs were selected) contributed significantly to expression variance across most markers in both PanCK + and PanCK- AOIs, suggesting the presence of spatial heterogeneity. Moreover, substantial residual variance was observed for most markers, indicating unmeasured sources of spatial, biological, or technical heterogeneity. To further explore this, we included AOI spatial coordinates (see **Methods**) into the LME models. Figure 4B illustrates pairwise comparisons of expression variance with (M1 model, indicated by the blue bar) and without the spatial variable (M0 model) for each marker. The pairwise comparison revealed that AOI spatial location accounted for additional variance in several markers, particularly in PanCK+ AOIs. Markers notably affected by spatial heterogeneity included TIM3, SMA, GZMB, CD34, and S100B in stroma-rich PanCK- segments, and CD8, TIM3, VISTA, HLA-DR, CD163, and PD-L1 in epithelium-rich PanCK+ segments.

### Multiplatform comparisons reveal intratumor heterogeneity in breast cancer intrinsic subtype classification

**Fig. 5:**
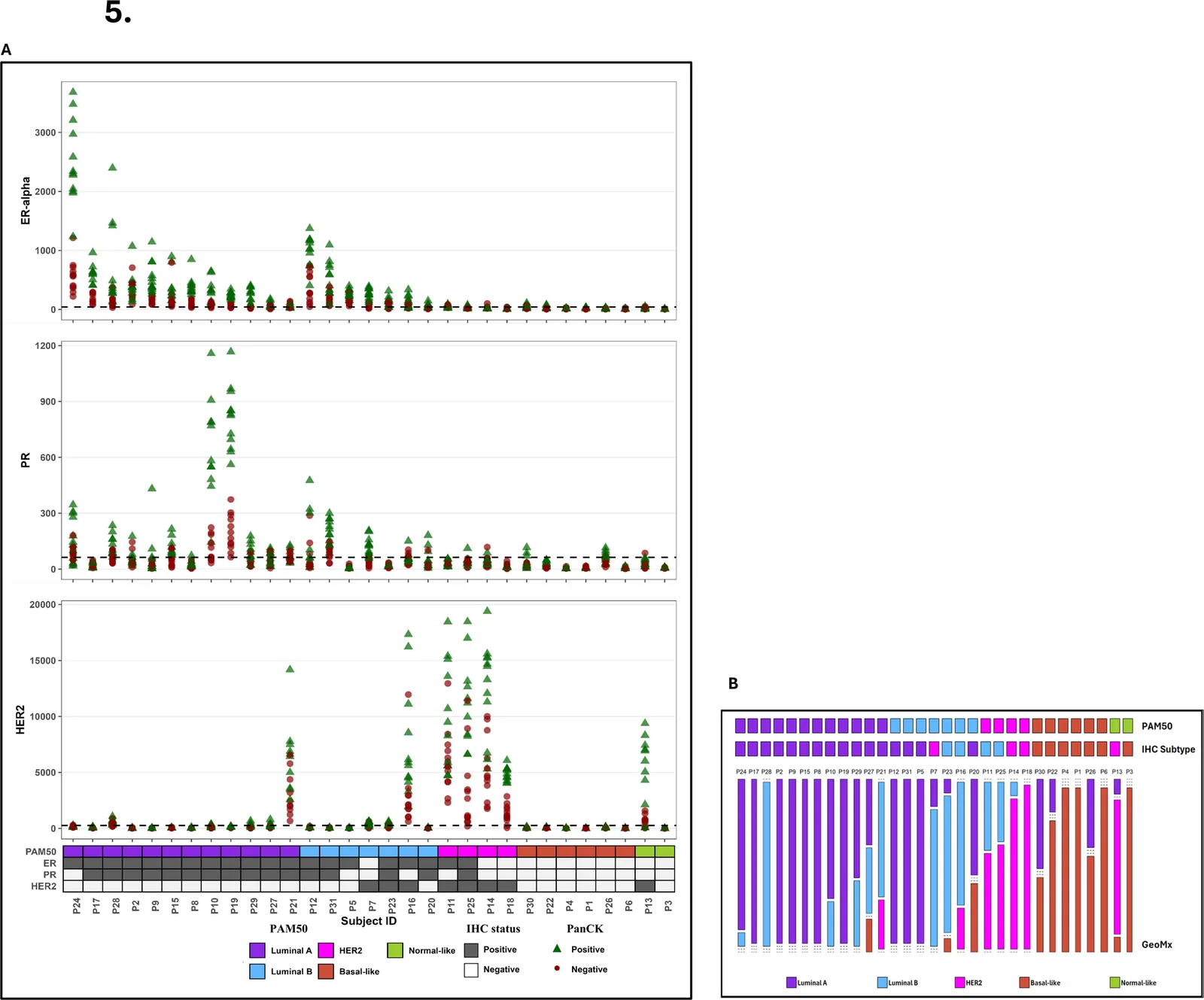
Multiplatform comparison of intratumor heterogeneity of intrinsic subtypes. Panel** A**: The distribution of estrogen receptor (ER), progesterone receptor (PR), and human epidermal growth factor receptor 2 (HER2) expression scores across AOIs by GeoMx compared to bulk PAM50 subtypes as well as immunohistochemical (IHC) scoring of ER, PR, and HER2. Panel** B**: Distribution of AOI-level intrinsic subtype across PanCK+ AOIs within each tumor. AOI-specific subtypes were assigned based on expression levels of ER-alpha, PR, and HER2 in each AOI using an optimal cut-off point that maximized the separation between ER + and ER− tumors. Subtypes were defined as follows: Luminal A: ER + and/or PR+, HER2−; Luminal B: ER + and/or PR+, HER2+; HER2-enriched: ER−, PR−, HER2+; and Triple Negative: ER−, PR−, HER2−

Breast cancer treatment decisions are often informed by molecular subtypes, defined by markers such as the immunohistochemical (IHC) expression of ER, PR, and HER2, or multigene-based expression signatures. To assess ITH of intrinsic subtypes, we compared the spatial expression levels of ER, PR, and HER2 across AOIs determined by GeoMx with whole-tissue section IHC data and bulk PAM50 subtypes by NanoString nCounter. As shown in Fig. 5A, ER and PR expression were uniformly low across all AOIs in non-luminal (HER2-enriched, basal-like, and normal-like) or in tumors that were ER-/PR- by whole-tissue section IHC. In contrast, luminal tumors or IHC ER+/PR+ tumors exhibited greater variability in ER and PR expression, with PanCK+ AOIs generally showing higher expression than PanCK- AOIs. All basal-like tumors demonstrated low ER, PR, and HER2 expression across all AOIs. The four HER2-enriched tumors showed high HER2 expression in most, but not all, AOIs. Notably, two tumors - one classified as luminal B (P16) and the other as normal-like (P13) by PAM50 – displayed high HER2 expression across AOIs and were also identified as HER2 + by IHC. Based on the evidence from all assays, the normal-like tumor may have been more accurately classified as HER2-enriched. Additionally, one tumor (P21), consistently classified as luminal A by both PAM50 and whole-tissue section IHC, showed high HER2 expression in most (especially PanCK+) AOIs, highlighting a potential misclassification by bulk approaches due to ITH.

We then assigned an intrinsic subtype to each AOI based on AOI-level ER, PR, and HER2 expression (see Methods), restricting to PanCK+ AOIs. Figure 5B illustrates the ITH of subtype assignments and compares AOI-level classifications with patient-level subtypes determined by NanoString bulk PAM50 and whole-tissue section IHC of ER, PR, and HER2. In general, AOI-level subtype assignments were consistent within a single tumor for most tumors, with some of the variations likely attributed to the natural overlap between certain subtypes, such as luminal A vs. luminal B or luminal B vs. HER2-enriched. However, three patients exhibited three subtypes, and another four patients had two contrasting subtypes (luminal A and TNBC) within a single tumor (Fig. 5B). Although in most of these patients the consistent subtype classification was observed between the two bulk approaches, which tended to call the predominant AOI-level subtype, the presence of multiple subtypes within a tumor could potentially influence treatment response. For example, for patients showing the intratumoral co-occurrence of luminal A and TNBC AOIs and both bulk assays classifying them as basal-like or TNBC, the presence of luminal A AOIs in these tumors raises the possibility that these patients could potentially benefit from endocrine therapies.

### Multiplatform comparisons reveal intratumor heterogeneity in breast tumor immune microenvironment

**Fig. 6:**
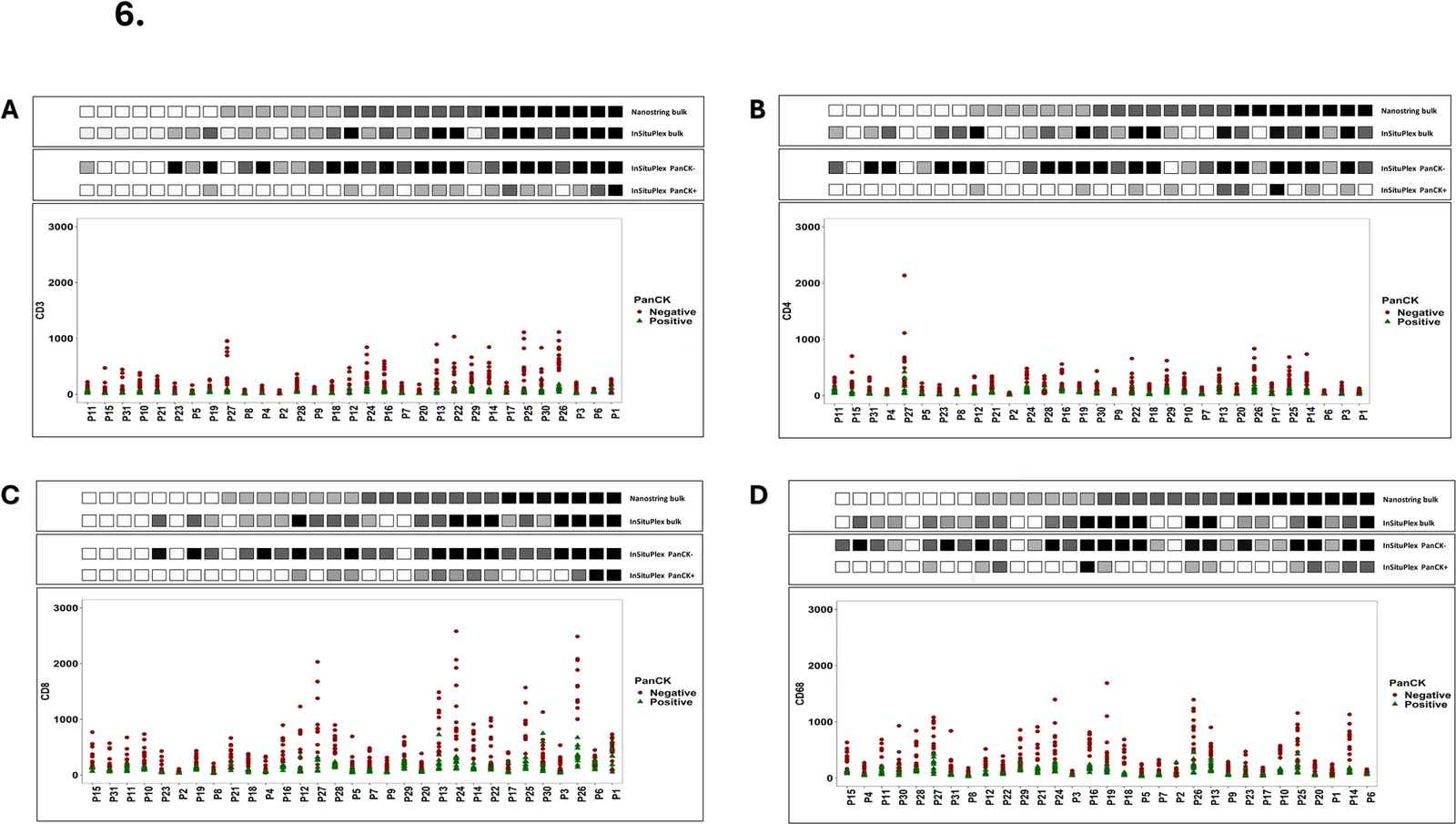
Multiplatform comparison of intratumor heterogeneity of immune cell marker expression. The distributions of CD3 (Panel A), CD4 (Panel B), CD8 (Panel C), and CD68 (Panel D) expression across AOIs within each tumor compared to two bulk approaches (NanoString bulk profiling and InSituPlex overall, categorized into quartiles) and PanCK compartmentalized expression data from InSituPlex staining using the same cutoff values determined from the overall distribution of each marker to generate quartiles

To assess the impact of immune marker ITH on tumor classification, we compared AOI-level GeoMx data with overall and compartmentalized expression data from InSituPlex staining, segmented into PanCK + and PanCK- regions, as well as bulk NanoString expression data. Six immune markers were analyzed across all three platforms: CD3, CD4, CD8, CD68, PD-1, and FOXP3. Among these, CD3, CD4, CD8, CD68 showed abundant expression, so we focused on these four markers for multi-platform comparisons. Due to differences in scales used by each platform, we categorized NanoString bulk expression and InSituPlex staining data into quartiles. For the InSituPlex staining data, we applied the same cutoff values, determined from the overall distribution of each marker to generate quartiles (Fig. 6).

The expression of T cell markers CD3 (Fig. 6A) and CD4 (Fig. 6B) was largely consistent between the two bulk assays (NanoString bulk and InSituPlex bulk) and generally aligned with results obtained from GeoMx. However, there were some notable exceptions. Some tumors with low expression based on both bulk approaches contained high-expressing AOIs, particularly in stroma-rich areas. Conversely, several tumors ranked among the highest expressers in both bulk assays, such as P26, showed low expression across most AOIs. This discrepancy may be due to sampling bias, where highly expressing areas were not selected for AOI analysis, but it also highlights significant ITH in these tumors. In contrast to CD3 and CD4, CD8 and CD68 markers demonstrated moderate to substantial ITH in their expression. For CD8 (Fig. 6C), patients with higher marker expression based on bulk approaches also tended to show correspondingly higher CD8 expression across multiple AOIs. However, a subset of patients with low CD8 expression based on bulk had several AOIs demonstrating high CD8 expression. The agreement between bulk and spatial approaches was less consistent for CD68 (Fig. 6D), which was characterized by substantial ITH for the majority of the patients for this marker. Notably, several tumors, such as P27, P24, and P26, exhibited extensive ITH across markers. For these tumors, both sampling-based approaches like tissue microarrays (TMAs) or ROI-based assays and bulk assays are likely to produce misleading and inconsistent measurements of immune markers. In low-resource settings, in situ assays combined with multi-region scoring could offer a more accurate yet practical alternative to bulk or single-cell assays, which are often costly and technically challenging.

## Discussion

Breast cancer treatment is commonly guided by intrinsic subtype classification, which is typically assigned at the patient-level by using bulk profiling approaches. The growing prominence of immunotherapies highlights the importance of molecular heterogeneity, not only in tumor cells but also within the immune TME, for informing treatment decisions and predicting outcomes [26]. The presence of spatial ITH poses challenges in accurately classifying patients when relying solely on bulk molecular measurements. Many spatial profiling methods, such as single-cell sequencing, are expensive and technically demanding, which limits their widespread applicability, particularly in low-resource settings. In this study, we investigated the spatial heterogeneity of intrinsic tumor and immune TME markers by comparing data from multiple spatial and bulk platforms in the same cohort of breast cancer patients from an underrepresented East African population.

Consistent with previous studies assessing ITH by analyzing multiple samples from a single breast tumor [27,28], we found that the expression of subtyping markers ER, PR, and HER2 showed high concordance between spatial and bulk analyses. Despite spatial heterogeneity, bulk approaches consistently achieved concordant subtype classification for most tumors. Notably, ER and PR showed uniformly low expression across AOIs among non-luminal tumors. However, in luminal tumors, a subset of patients showed highly variable ER and PR expression across AOIs, leading to the co-occurrence of multiple subtypes within a single patient (Fig. 2C). This raises an important question regarding whether these patients might benefit from combined therapies that account for ITH and corresponding tumor molecular diversity. Previous studies using luminal tumor xenografts identified a subpopulation of ER-PR-CK5+ (luminobasal) cells that were resistant to endocrine therapies but were suppressed by EGFR inhibitor [29]. Dual application of endocrine therapy and EGFR inhibitors demonstrated efficacy for heterogeneous tumors with both luminal and basal cells [30]. Additionally, Blows et al. demonstrated that luminal A tumors expressing basal markers (CK5 or CK5/6, EGFR) had worse prognosis than luminal A tumors negative for basal markers in a large consortium analysis including > 10,000 invasive breast cancer cases [31]. On their part, Li et al. demonstrated that intra-tumoral HER2 heterogeneity impacted treatment-induced transcriptomic changes and response to HER2-targeting antibodies [32]. In their study, HER2 heterogeneous tumors showed minimal transcriptomic changes and limited response to anti-HER2 antibody treatment when compared to HER2 non-heterogeneous tumors [32]. While the small sample size in our study limited our ability to evaluate treatment responses or conduct survival analyses, we propose that identifying ITH through multi-region IHC scoring could be incorporated into and evaluated within clinical trials.

Our findings align with recent studies emphasizing the importance of spatial context in understanding the TME and its influence on tumor behavior and treatment response. For instance, Chen et al. demonstrated that multi-dimensional immune signatures, incorporating immune marker density and spatial locations, improved predictions of response to immune checkpoint blockades (ICBs) in gastric cancer patients [33]. Similarly, Hammerl et al. found that spatial immunophenotypes were significantly associated with prognosis in TNBC patients and that a gene classifier of spatial phenotypes predicted response to anti-PD1 treatment [34]. In our analysis, most immune markers exhibited significant spatial heterogeneity within the TME. As expected, tissue composition (epithelium-rich or stroma-rich) was the major contributor to ITH in immune markers, with most markers showing higher expression in stroma-rich regions, particularly when these regions were within stroma-rich neighborhoods. Interestingly, some markers exhibited expression patterns that differed between AOI and neighborhood morphology, suggesting an extensive effect of the local cellular ecosystem. Further, some markers, such as TIM3, demonstrated significant spatial variations even after adjusting for common location-associated variables such as AOI/neighborhood morphology, tissue size, and AOI phenotype. Pathological review of TIM3 staining did not reveal any technical concerns, suggesting that ITH in these markers may be driven by unmeasured confounders or intrinsic location-based variability. Consistently, we found that tissue area significantly contributed to marker expression variance for most markers, raising concerns that evaluations based on tissue microarray (TMA) cores may lead to misclassifications.

Our variance decomposition analysis further revealed that both measured and unmeasured factors contribute substantially to intertumoral and intratumoral heterogeneity across protein markers. Patient-to-patient variation accounted for a large proportion of total variance, underscoring strong intertumoral heterogeneity. Among measured factors, intrinsic subtype (PAM50) emerged as the dominant determinant of marker variability in both epithelial- and stroma-rich compartments, influencing not only classical subtyping markers (ER, PR, HER2) but also immune and signaling markers such as IDO1, S100B, PTEN, and BCL-2. Morphologic and neighborhood-level features, particularly in stroma-rich AOIs, further shaped the expression of several immune and stromal markers, reflecting local TME influences. Spatial position within the tissue section also contributed additional variance, highlighting a layer of spatial heterogeneity not captured by morphologic or molecular classification alone. Notably, a substantial proportion of residual variance persisted even after accounting for all known covariates, indicating that unmeasured biological or spatial factors—such as microregional cell–cell interactions or technical variation in tissue sampling—may further drive heterogeneity. Together, these results emphasize that breast cancer heterogeneity arises from multiple, interacting levels of variation spanning tumor-intrinsic, microenvironmental, and spatial domains.

The unique design of our study allowed us to compare the expression of several key immune markers across different platforms, including spatial (AOI-based), semi-spatial (whole-section staining annotated by PanCK), and bulk (whole tissue mass) profiling within the same set of patient samples. For markers with low (CD3 and CD4) or moderate (CD8) ITH, rankings across different patients were generally consistent between the two bulk platforms (NanoString and InSituPlex) and between bulk and spatial analyses. As expected, expression of these immune markers was significantly higher in PanCK- compared to PanCK+ regions, as demonstrated in both GeoMx and InSituPlex PanCK-segmented analyses. For these markers, bulk or PanCK-segmented analyses may provide reasonable estimates of expression levels. However, our data indicated high ITH and poor agreement across platforms for CD68, a macrophage marker with key roles in cancer progression, immune evasion, and response to therapy [35]. CD68 + macrophages have been shown to cluster in specific regions such as the tumor leading edge, perivascular niches, or hypoxic areas [36]. This spatial heterogeneity may influence tumor-immune interactions and impact responses to immunotherapies, chemotherapy, or macrophage-targeting therapies (e.g., CSF1R inhibitors) [37].

Across the examined markers, especially TME markers, we observed marked differences between bulk and spatial platforms in several patients. In some cases, these discrepancies were due to extensive ITH, where bulk measurements reflected the overall average. In other cases, sampling-based approaches, including ROI selection and TMAs, may have led to incomplete or biased regional representation. Under both scenarios, small regions with distinct immune expression profiles may be missed by either bulk or ROI-based approaches, potentially affecting therapeutic predictions and responses. Our findings underscore the necessity of considering both tumor and TME heterogeneity when determining optimal therapeutic strategies, including the use of ICBs.

The major limitation of our study is the small sample size, which precluded an assessment of ITH’s impact on treatment responses and patient outcomes. Additionally, although the included patients were selected to be broadly comparable to the larger Kenyan source cohort with respect to ER-positivity distribution and major clinicopathologic characteristics, the analytic cohort was restricted to tumors with sufficient tissue quality, suitable morphology for ROI selection, and complete data across all three platforms. Therefore, selection bias related to tissue availability, tissue quality, and platform-specific QC cannot be fully excluded. In addition, both the analytic cohort and the larger source cohort were hospital-based and may not be representative of the general breast cancer population in Kenya or other African countries. Furthermore, although GeoMx-measured PanCK expression provided a quantitative approach to standardize ROI morphology across patients and tissue sections, the initial ROI selection was pathologist-guided and may not have captured the full complexity of tissue morphology, immune infiltration, and spatial marker gradients across the entire tumor section. Future studies using whole-slide spatial profiling or independently selected replicate ROI sets would help quantify the sensitivity of spatial heterogeneity estimates to ROI selection. Finally, the variance of expression levels for low-expressing markers may not have been estimated accurately in the LME models. Nevertheless, this study represents the first comprehensive spatial TME analysis of breast tumors from a sub-Saharan population. We found that most tumors assessed in our study demonstrated varying degrees of ITH, with about one third displaying substantial ITH. Since ITH presents a significant challenge for tumor phenotyping and treatment response assessment, and both bulk and sampling-based approaches have inherent limitations, a hybrid approach that integrates in situ whole-slide scoring with multi-region digital scoring to better account for ITH in clinical settings might be warranted.

## Supplementary information

Below is the link to the electronic supplementary material.

Supplementary Material 1

Supplementary Material 2

## Data Availability

Data and images analyzed in the current study can be obtained from the corresponding author on reasonable request.
