# Supplementary material for: Spatially dissecting drivers of inter- and intra-tumoral heterogeneity in Kenyan breast cancer
Source: Breast Cancer Res. 2026 Jul 29;28:182. doi: 10.1186/s13058-026-02347-z (PMC13700304; doi:10.1186/s13058-026-02347-z)
Supplement: Supplementary file 1 [file 13058_2026_2347_MOESM1_ESM.docx]

**Supplementary Figure 1**: The signal to noise ratio (SNR) of each protein marker using the geometric mean of the background immunoglobulin G (IgG) controls.

**Supplementary Figure 2**: Differential marker expression by patient and AOI-level characteristics.

**Supplementary Figure 3:** Marker expression by AOI-phenotype (PanCK segmentation of AOIs) and neighborhood morphology (PanCK+/PanCK- status in neighborhood surrounding AOIs). AOI neighborhood was defined by expanding 500 µm from the primary AOI. Using the Area and CytoNuclear modules for PanCK, the amount of PanCK was quantified for each AOI neighborhood.
